# First Molecular Identification of Symbiotic Archaea in a Sponge Collected from the Persian Gulf, Iran

**DOI:** 10.2174/1874285801812010323

**Published:** 2018-10-17

**Authors:** Akram Najafi, Maryam Moradinasab, Mohammad Seyedabadi, Mohammad A. Haghighi, Iraj Nabipour

**Affiliations:** 1 The Persian Gulf Marine Biotechnology Research Center, Bushehr University of Medical Sciences, Bushehr, Iran; 2 The Persian Gulf Tropical Medicine Research Center, Bushehr University of Medical Sciences, Bushehr, Iran; 3 Department of Pharmacology, School of Medicine, Bushehr University of Medical Sciences, Bushehr, Iran; 4 Department of Microbiology and Parasitology, Faculty of Medicine, Bushehr University of Medical Sciences, Bushehr, Iran

**Keywords:** Porifera, Uncultured Archaea, Symbiosis, *Chondrilla australiensis*, Persian Gulf, Taxonomy

## Abstract

**Background:**

Marine sponges are associated with numerically vast and phylogenetically diverse microbial communities at different geographical locations. However, little is known about the archaeal diversity of sponges in the Persian Gulf. The present study was aimed to identify the symbiotic archaea with a sponge species gathered from the Persian Gulf, Iran.

**Methods:**

Sponge sample was collected from a depth of 3 m offshore Bushehr, Persian Gulf, Iran. Metagenomic DNA was extracted using a hexadecyl trimethyl ammonium bromide (CTAB) method. The COI mtDNA marker was used for molecular taxonomy identification of sponge sample. Also, symbiotic archaea were identified using the culture-independent analysis of the *16S rRNA* gene and PCR- cloning.

**Results:**

In this study, analysis of multilocus DNA marker and morphological characteristics revealed that the sponge species belonged to *Chondrilla australiensis* isolate PG_BU4. PCR cloning and sequencing showed that all of the sequences of archaeal *16S rRNA* gene libraries clustered into the uncultured archaeal group.

**Conclusion:**

The present study is the first report of the presence of the genus of *Chondrilla* in the Persian Gulf. Traditional taxonomy methods, when used along with molecular techniques, could play a significant role in the accurate taxonomy of sponges. Also, the uncultured archaea may promise a potential source for bioactive compounds. Further functional studies are needed to explore the role of the sponge-associated uncultured archaea as a part of the marine symbiosis.

## 1 INTRODUCTION

Marine sponges are one of the oldest and most important multicellular animals (metazoans) and reef builders in benthic communities’ worldwide [1, 2]. In the past decade, many studies have focused on the sponge-associated microbial communities because of their benefit the hosts with different functional roles [3-6]. These microorganisms constitute up to 40-60% of the total sponge biomass and may affect the sponge survival in the marine through producing bioactive secondary metabolites, photosynthesis, nitrogen and sulfur fixation,* etc*. [1, 4, 5, 7].

Traditionally, the taxonomy of marine sponges is defined based on morphological features, using some diagnostic characters. Because of the limitations of informative characters and a wide range of phenotypic plasticity, the sponge classifications have been hindered [8]. Currently, molecular data have been particularly used in sponge phylogenetic analysis, with the variable 28S rDNA region, the highly variable Internal Transcribed Spacer (ITS) region, and mitochondrial DNA (cytochrome oxidase subunit 1 (COI)) [9].

Archaea are comprised the third phylogenetic domain of life and their presence in sponge larvae reveals a tight relationship between archaeal symbionts and the sponge host phylogeny [10, 11]. However, environmental factors including seasonal changes in seawaters or geographical distribution of sponges may also influence the relationship [12].

Within the past two decades, culture-independent techniques such as *16S rRNA* gene-based clone library have led to a deeper understanding of the microbial diversity, and a wide distribution of mostly uncultured archaea in different species sponges [13-15]. More than 20,000 archaeal *16S rRNA* gene sequences have been presented from environmental studies, extending the known groups and introducing of novel lineages [13].

To date, more than 25 different bacterial phyla and 2 archaeal lineages have been reported from sponge species from different geographic locations [2, 11, 16]. But little is known from the Persian Gulf. The Persian Gulf, is a unique and greatly underexplored marine ecosystem containing 55 sponge genera recorded [17]. To our knowledge there is no research reported on the archaeal communities of sponges in Iran.

The present study was aimed to identify the symbiotic archaea with a sponge species (*Chondrilla australiensis* isolate PG_BU4) collected from the Persian Gulf, Iran.

## 2 MATERIALS AND METHODS

### 2.1 Sampling and Identification of Sponge

Sponge sampling was performed in July 2016 at a depth of 3 m offshore Bushehr, Persian Gulf, Iran (GPS: 28°58'54.4”N 50°49'27.8”E) by SCUBA diving. Sponge sample was raised with 0.22-μm-membrane-filtered seawater (FSW) to remove exogenous materials and loosely attached microbes. Sample was placed in sterile plastic Ziploc bags and immediately transported to the laboratory on dry ice and then frozen at −80°C [18].

### 2.2 Morphological Identification of Sponge

In this study, morphological and spicule examination was carried out by Dr. Yusheng M. Huang (National Penghu University of Science and Technology, Taiwan). The skeleton and spicule slide were examined using a compound light microscope (Leica DM2500 with MC170 HD Camera & SW Kit) and photographed and measured using Leica Application Suite ver. 4.11. The morphological features of sponge spicules, skeletons, and choanocyte chambers were described using Boury-Esnault and Rützler 1997 and further identified according to the key, Systema Porifera: A guide to the Classification of Sponges [8] and references provided by the World Porifera Database [19].

### 2.3 Metagenomic DNA Extraction

Sponge sample was washed with sterilized seawater and cut into small pieces (about 1 cm^3^). Sponge tissues were ground to fine powder under liquid N_2_ using a sterile pestle and mortar [10, 18]. Metagenomic DNA was extracted using a hexadecyltrimethylammonium bromide (CTAB) according to Schmitt *et al*. 2012 method [20]. Metagenomic DNA was qualified by electrophoresis on 1% agarose gel. The quantitative assessment of the isolated DNA was carried out using a NanoDrop ND-100 device (Thermo Fisher, USA) and then stored at −20 °C until use.

### 2.4 Sponge COI gene PCR

In this study, a mitochondrial gene, cytochrome oxidase subunit 1 (COI) was amplified. Primer sequences are shown in Table **1**. Briefly, a single reaction mixture containing 2.5 µl of 10x buffer (supplied with Taq polymerase), 1 µl of DNA (approximately 100 ng/µl), 0.5 µl of each appropriate primer (10 mM) (Shanghai Generay Biotech Co., Ltd), 3 µl dNTPs (1 mM), 1 µl of MgCl_2_, 0.25 µl of Taq DNA polymerase (Fermentase, Lithuania) in a total volume of 25 µl [21]. PCR amplifications were carried out on a thermal cycler PeQlab, peqSTAR 96X Universal Gradient, Germany under the following conditions: 94°C for 5 min; followed by 35 cycles of 94°C for 40 s; 58°C for 30 s; 72°C for 40 s; followed by 72°C for 5 min. PCR products were purified and sequenced by Macrogen Inc (Seoul, Korea).

### 2.5 Archaea 16S rRNA gene PCR

Approximately 1000 to 1300 bp of the *16S rRNA* gene was amplified by PCR using the universal archaeal primers (Table **1**). PCR consisted of a reaction of 25 µl with: 2.5 µl of 10x buffer, 1 µl of DNA (approximately 100 ng/µl), 1 µl of each forward and reverse primers (10 mM) (Shanghai Generay Biotech Co., Ltd), 0.5 µl of dNTPs (1 mM), 1 µl of MgCl_2_, 0.5 µl of Taq DNA polymerase (Fermentase, Lithuania). Thermal cycling was initiated with denaturation at 94◦C for 5 min, followed by 30 cycles of 15 s at 94◦C, 30 s at 51 and 72◦C for 1 min and a final extension step for 7 min at 72◦C [23]. PCR products were purified using the NucleoSpin® Gel and PCR Clean-up (Macherey-Nagel, Germany) and quantified using NanoDrop ND-100 device (Thermo Fisher, USA).

### 2.6 Cloning of PCR Products and DNA Sequence Analysis

*16S rRNA* gene libraries were constructed in JM109 *Escherichia coli* competent cell using a pGEM®-T easy cloning kit (Promega, USA) following the manufacturers' instructions. After a blue-white screening, presumptive recombinant white colonies were randomly picked, subcultured onto LB agar containing ampicillin 100 mg ml^-1^, X-Gal 80 mg ml^-1^, and IPTG 0.5 mM. Individual clones were PCR-screened using universal primers and 10 clones with approximately 1000 to 1300 bp inserts were recovered from samples. Plasmid DNA was purified using the NucleoSpin® Plasmid and NucleoBond® Xtra Midi kits (Macherey-Nagel, Germany), according to the manufacturer, from the selected colonies [24]. Inserts of representative plasmids were sent to Macrogen Inc (Seoul, Korea) for two reads sequencing using both vector primers (pUC/M13F: 5′-d(GTTTTCCCAGTCACGAC)-3′ and pUC/M13R: 5′-d(CAGGAAACAGCTATGAC)-3′) and primers used to generate the corresponding library.

### 2.7 Phylogenetic Analysis

All nucleotide sequences obtained for *16S rRNA* and COI genes phylogenetic analysis and their top BLASTN hits (GenBank database) were manually edited and aligned using the ClustalW (v. 1.81) program. Consequently, two alignment datasets were generated: 588 sites of 5 sequences (COI for sponges) and 1391 sites of 20 sequences (*16S rRNA* for archaea). The alignment dataset was analyzed using Maximum Likelihood (ML) and Neighbor-Joining (NJ) methods. Maximum likelihood and neighbor-joining analyses were conducted using the MEGA7 (v. 7.0.18) program, with 1000 bootstrap resamplings.

## 3 RESULTS

### 3.1 Sponge Identification

**Fig. (1):**
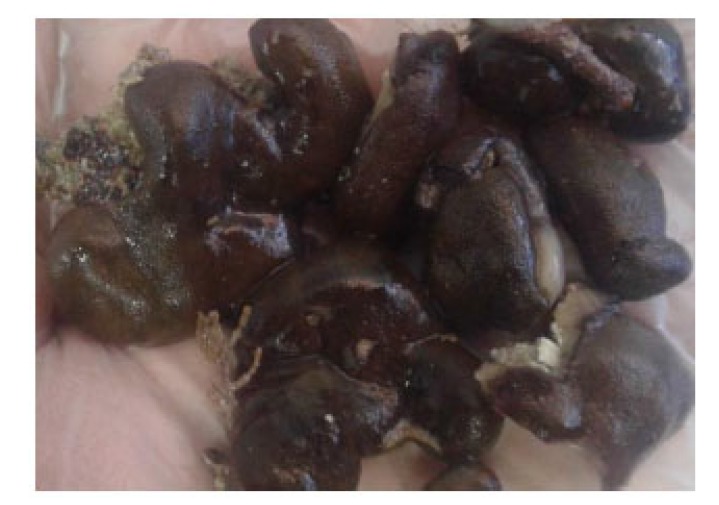
A *Chondrilla australiensis* yellow-brown color morph.

In the present study, the collected sponge was identified as *Chondrilla australiensis* by Dr. Yusheng M. Huang (National Penghu University of Science and Technology, Taiwan), based on morphological characteristics. The sponge specimen was massive and sub-globose. The sponge had a combination of yellow-brown and greenish to dark brown colors, with shiny, smooth, and slippery surface and thickly encrusting (Fig. **1**).

**Fig. (2):**
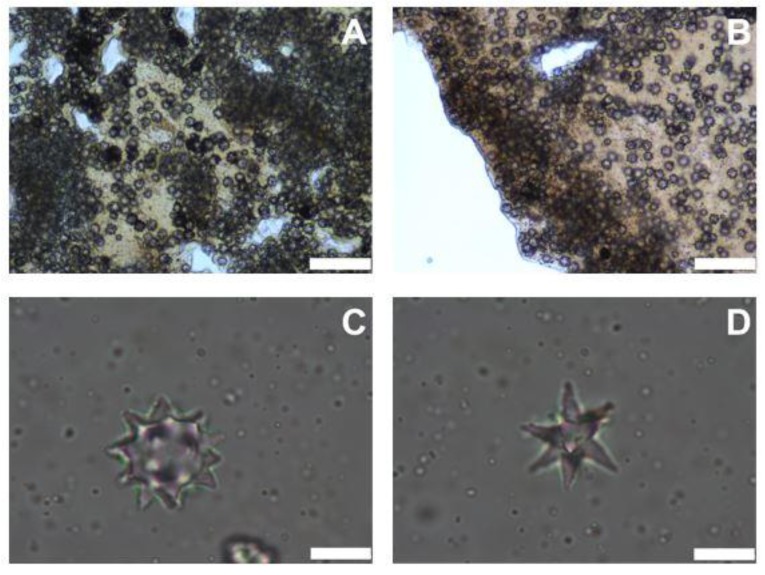
Light micrograph of *Chondrilla australiensis* skeleton. **A**) Ectosomal skeleton (scale bar 100 μm), **B**) Choanosomal skeleton (scale bar 100 μm), **C**) Spheraster (scale bar 10 μm), **D**) Speroxyaster (scale bar 10 μm).

Ectosome had a layer of dense spherasters (Fig. **2A**). The cortex was about 335-804 μm thick with a cuticle of about 74-149 μm (Fig. **2B**). It was composed of two layers; the upper one had a dense sheet of sherulous cells and spherasters (Fig. **2C**), whereas the internal one was made of scattered spherulous cells and spheraters. Also, Spheraster was 20-23-26 μm and Speroxyaster about 20 μm (Fig. **2D**).

### 3.2 Phylogeny of COI

**Fig. (3):**
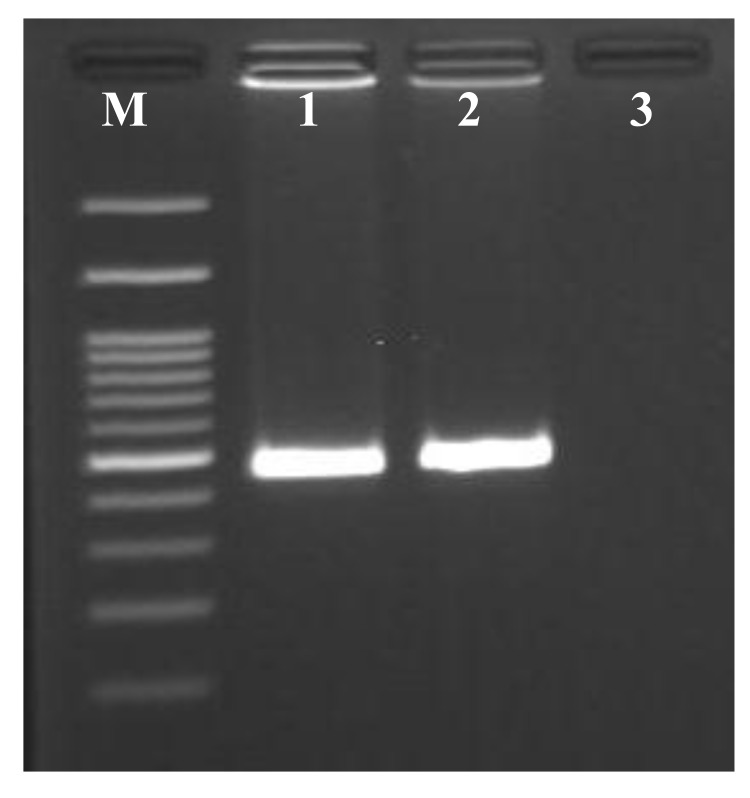
PCR amplification of mit *COI* gene in the sponge sample. M: 100 bp DNA ladder, Lanes 1 and 2: amplified mit *COI* gene, Lanes 3: C- .

PCR of metagenomic DNA isolated from sponge *Chondrilla australiensis* tissue samples using COI primer specific yielded a band with the expected size of 500 bp (Fig. **3**). In the present study, the most appropriate ML model selected was Tamura 3- parameter model with the uniform rate among sites (T92). The phylogenetic tree for the mitochondrial COI composed of major genu-level clades, corresponding to the *Chondrilla*. This genus formed a clade with generally high bootstrap value in phylogenetic analysis (ML=100% and NJ= 89%). Within the *Chondrilla* clade, the sequence was similar to a previously reported sequence of *Chondrilla australiensis* voucher MCZ from the United States of America (JX999064).

### 3.3 Phylogenetic Analysis of Archaea

**Fig. (4):**
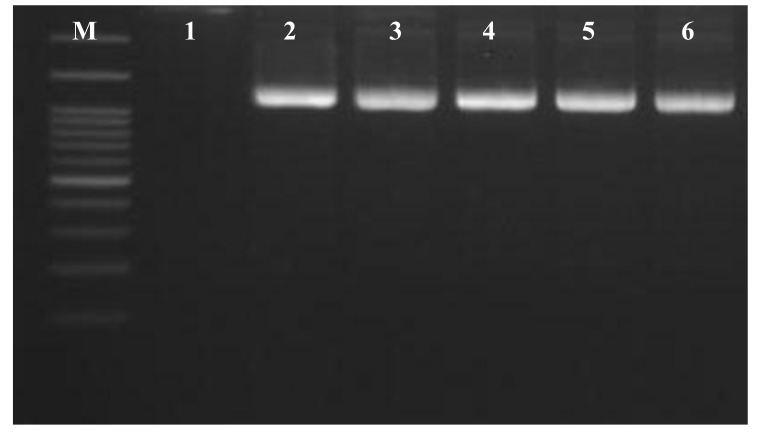
PCR amplification of *16S rRNA* gene in the studied samples. M: 100 bp DNA ladder, Lane 1: C-, Lanes 2 to 6: amplified universal archaeal *16S rRNA* gene.

In this study, PCR of total DNA isolated from *Chondrilla australiensis* samples using the universal archaeal primers showed a band of expected size of 1300 bp (Fig. **4**).

The PCR products were cloned into the pGEM®-T easy vector, producing 5 clone libraries with 20-50 white colonies each. 25 of these clones were run with M13 and universal primers. Of these, only 5 clones were amplified and subsequently sequenced. *16S rRNA* sequence analysis showed that these five “supposable” Archaeal clones are located in the uncultured archaeon group. Phylogenetic analysis of the *16S rRNA* sequence by ML and NJ analyses produced trees with nearly similar branching patterns. The best-fit ML model selected was Tamura–Nei with a gamma distribution of substitution rates among sites (TN93+G). Maximum likelihood and neighbour-joining analyses presented similarly well-resolved phylogenies.

**Fig. (5):**
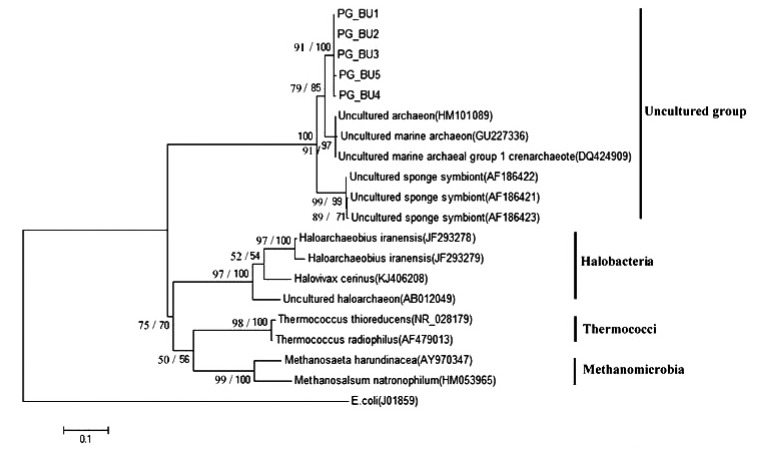
Phylogenetic analysis of *16S rRNA* gene sequences from uncultured Archaea. The tree was constructed using the maximum likelihood method and bootstrap resampled 1000 times. Bootstrap values greater than 50% are indicated on the internal nodes. Scale bar: 0.1 substitutions per nucleotide position. Genbank accession numbers are noted after the name of the strains.

In the present study, the phylogenetic tree for the *16S rRNA* gene composed of a major clade, corresponding to the uncultured group. Within this group, the PG_BU, Uncultured marine archaeon and Uncultured sponge symbiont formed a very well-supported clade in both ML (100%) and NJ (99%) analyses (Fig. **5**). Within this clade, the PG_BU and uncultured marine archaeon formed a clade supported by ML and NJ analyses (85% and 79%, respectively).

Uncultured sponge symbiont formed a very well-supported monophyletic clade in all analyses (ML: 99%, NJ: 99%). Also well-supported class-level clades were recovered for Halobacteria (100%, 97%), Thermococci (100%, 98%), and Methanomicrobia (100%, 99%). In this study, the *16S rRNA* gene sequence from *Escherichia coli* (J01859) was considered as outgroup.

### 3.4 Nucleotide Sequence Accession Numbers

The mt COI gene sequence for the sponge sample was deposited in GenBank under accession number LC372995. While *16S rRNA* gene sequences for the archaea were deposited under accession numbers LC216351 to LC216355.

## 4 DISCUSSION

To our knowledge, it is the first study focusing on the molecular taxonomy of sponge using multilocus DNA marker and also identification of symbiotic archaea through the culture-independent analysis of the *16S rRNA* gene in Iran.

The analysis of multilocus DNA marker along with the morphological features revealed that the sponge species belonged to *Chondrilla australiensis* isolate PG_BU4. This is the first report of the presence of the genus of *Chondrilla* in the Persian Gulf. Different studies have considered *Chondrilla* as one of the most distributed genera with about 17 species at different geographical locations [19, 25-29]. This genus is characterized based on the presence of siliceous spicules of the aster type alone, abounding with the cortex and surrounding the canals. This finding is in accordance with the morphological features of the studied sponge, here. Traditional taxonomy methods, when used along with DNA barcoding or other molecular techniques, may play an important role in the accurate taxonomy of sponges with very few phenotypical variations [29].

Archaeal communities are present in a wide range of sponges from different ecosystems under varying marine environmental conditions. Diversity, composition, functional ecology and distribution of symbiotic archaea in marine sponges have been investigated in different studies [5, 12, 30-34].

Based on these studies, the archaeal symbiont in marine sponges varied depending on host biogeography. In another word, the specific geographical location of sponges has a great influence on their specific archaeal signatures. In addition, structure and composition of sponge-associated archaeal communities can be changed in response to marine pollution [12]. The Persian Gulf as a semi-closed marine environment with specific physicochemical characteristics and high levels of oil pollution and petroleum wastes may have its unique sponge-associated archaeal composition.

The current work as the first investigation on the archaeal community of the Persian Gulf showed that *Chondrilla australiensis* harbors distinct uncultured archaea. In this study, the sequence from the *Chondrilla australiensis* PG_BU specimen formed a well-supported clade (ML=85%, NJ=79%) with uncultured marine archaeon from USA [33], South China Sea [35], and also with uncultured sponge symbiont from Western Caroline Islands, Palau [36].

Since the previous decade, detection and molecular analysis of uncultured archaea *16S rRNA* gene sequences from environmental sources have greatly surpassed several orders of magnitude above those reported cultured counterparts [37]. It has been suggested that most of the environmental *16S rRNA* sequences of the high taxa will be introduced in the next several years [38]. Therefore, other biotypes and non-host environmental sources from the Persian Gulf have this potential to discover novel archaeal lineages using molecular analysis. Although currently, no laboratory-cultivated strains for the majority of sponge associate archaea are available, cultivation of uncultured archaea with high bioactive compounds and biotechnological potentials from sponge sources will be accessible in the near future. The cultivability of (previously uncultured) bacterial genera from three Mediterranean sponges was reported very recently [39].

Although the stability of the sponge-archaea associations could not be inferred in the current study, consistent association and host- specificity of symbiotic archaea in marine sponges have been reported in different studies [5, 31]. It has also been suggested that archaeal community composition and symbiont-sponge interactions have evolved in parallel from many millions of years ago [33]. As a limitation of the study, we did not assess the composition of archaea in non-sponge hosts or non-host environment including seawater column and sediment.

The accumulated knowledge from genes and genomes of uncultivated environmental archaea provided insights about the potential metabolic capabilities of some of these uncultivated archaea [40, 41]. Recently, the bioinformatics and functional insights into the lifestyle of symbiotic uncultured bacterial product factories in marine sponges were presented [42]. By using genomic, (meta) proteomic and chemical methods, it is possible to gain some metabolic insights into the uncultured archaea that we have found in the current study.

The members of sponge symbiotic microbial community may be involved in providing food and chemical defense molecules for sponges and protect them from ultraviolet light. Archaea play a major role in nitrogen metabolism of sponges and ammonia oxidation [5]. It is generally accepted that sponge-associated microbes including the archaeal members are the main producers of bioactive and biochemical metabolites that have been reported from marine sponges [32]. Hence, from a biotechnological viewpoint, the identification of sponge-associated archaea that may produce bioactive compounds with potential pharmaceutical applications is a very interesting research field [20].

## CONCLUSION

The molecular identification of symbiotic archaea in a marine sponge collected from the Persian Gulf may promise a potential source for bioactive compounds with a range of activities including antibacterial properties against vectors of human diseases.

## Figures and Tables

**Table 1:** Primer sequences used for identification of sponge and Archaea.

**Organism**	**Primers**	**Sequences**	**Size (bp)**	**Reference**
**Sponge**	LCO1490 F	5′- GGTCAACAAATCATAAAGATATTGG- 3′	500	[22]
	HCO2198 R	5′- TAAACTTCAGGGTGACCAAAAAATCA- 3′		
**Archaea**	21F	5′- TTCCGGTTGATCCYGCCGGA- 3′	1300	[23]
	1492R	5′- GGTTACCTTGTTACGACTT- 3′		
